# Key Player in Cardiac Hypertrophy, Emphasizing the Role of Toll-Like Receptor 4

**DOI:** 10.3389/fcvm.2020.579036

**Published:** 2020-11-26

**Authors:** Zheng Xiao, Bin Kong, Hongjie Yang, Chang Dai, Jin Fang, Tianyou Qin, He Huang

**Affiliations:** ^1^Department of Cardiology, Renmin Hospital of Wuhan University, Wuhan, China; ^2^Cardiovascular Research Institute of Wuhan University, Wuhan, China; ^3^Hubei Key Laboratory of Cardiology, Wuhan, China

**Keywords:** Toll-like receptor 4, inflammation, cardiac hypertrophy, signaling pathway, immune response

## Abstract

Toll-like receptor 4 (TLR4), a key pattern recognition receptor, initiates the innate immune response and leads to chronic and acute inflammation. In the past decades, accumulating evidence has implicated TLR4-mediated inflammatory response in regulation of myocardium hypertrophic remodeling, indicating that regulation of the TLR4 signaling pathway may be an effective strategy for managing cardiac hypertrophy's pathophysiology. Given TLR4's significance, it is imperative to review the molecular mechanisms and roles underlying TLR4 signaling in cardiac hypertrophy. Here, we comprehensively review the current knowledge of TLR4-mediated inflammatory response and its interaction ligands and co-receptors, as well as activation of various intracellular signaling. We also describe the associated roles in promoting immune cell infiltration and inflammatory mediator secretion, that ultimately cause cardiac hypertrophy. Finally, we provide examples of some of the most promising drugs and new technologies that have the potential to attenuate TLR4-mediated inflammatory response and prevent or reverse the ominous cardiac hypertrophy outcomes.

## Introduction

The heart is the pump that maintains blood circulation. It drives blood through the blood vessels into various organs to supply oxygen and nutrients needed by the body's cells. Since adult cardiomyocytes lack regenerative ability, the heart often undergoes enlargement in cardiomyocytes size and thickening of ventricular walls following pre- or afterload increases (1). Cardiomyocytes respond to a series of stimuli that are defined as cardiac hypertrophy. There are two types of hypertrophy: physiological and pathological. Physiological cardiac hypertrophy is an adaptive change of the myocardium that occurs under hypertrophic stimuli, such as exercise training or pregnancy. However, in the presence of chronic stimulation conditions such as hypertension, valvular heart diseases, myocardial infarction, and neuro-hormones, this adaptive change can evolve into a maladaptive state, leading to pathological cardiac hypertrophy, which ultimately predisposes to heart failure (1, 2). Recent research evidence indicates that pathological differs significantly from physiological hypertrophy in terms of the underlying molecular mechanisms. Particularly, pathological cardiac hypertrophy is closely associated with chronic inflammation, which is accompanied by increased inflammatory cytokines (1–3). Thus, unraveling inflammatory signals that may regulate cardiac hypertrophy has significant implications.

Toll-like receptors (TLRs) are a family of pattern recognition receptors (PRRs) that recognize pathogen associated molecular patterns (PAMPs) derived from various microorganisms, including bacteria, viruses, and pathogens. In mammalian, these PAMPs initiate innate immune and inflammatory response (4, 5). Additionally, TLRs are involved in identifying damaged associated molecular patterns (DAMPs), which are released by host cells following cell or tissue damage (5, 6). Meanwhile, TLRs have emerged as crucial regulators in cardiovascular diseases, and their specific roles have been characterized. Particularly, Toll-like receptor 4 (TLR4), a major member of the TLR family, has been shown to be an inflammatory protein. For example, previous reviews have reported its therapeutic efficacy (4, 7, 8), and recently, it was found to be a significant inflammatory molecule that plays a central role in pathogenesis of hypertension and cardiac hypertrophy (9).

Functionally, TLR4 activation initiates the signaling cascade giving rise to a large repertoire of inflammatory cytokines, that reportedly regulate inflammatory responses and progression of cardiac hypertrophy. In this review, we discuss TLR4-related inflammatory signaling and their intricate molecular mechanism in cardiac hypertrophy, such as ligands, co-receptors, inflammatory pathways, immune cells, and inflammatory mediators.

## Signal Transduction Mechanism of TLR4

Human TLR4, a single-pass transmembrane protein that can induce inflammatory response by binding to PAMPs or DAMPs, was the first ever characterized mammalian TLRs. Specifically, PAMPs and DAMPs act as exogenous or endogenous ligands that activate TLR4 signaling and induce inflammatory response across many pathological processes (4, 10). A closer illumination of TLR4's structure and function indicates that it forms complex with its co-receptors before binding to specific ligands, which is an indispensable process in the initiation of inflammatory response. Particularly, lipopolysaccharide (LPS) is the most extensively studied PAMP. Previous studies have shown that this classical ligand binds to LPS binding protein (LBP), and the LBP/LPS complex attaches to another protein known as cluster of differentiation 14 (CD14), which catalyzes LPS transfer to another complex pre-formed by TLR4 and its co-receptor myeloid differentiation protein 2 (MD2). This reaction subsequently forms the LPS/TLR4/MD2 complex that triggers a series of inflammatory signaling pathways (11, 12). On the cell membrane, TLR4 binds to its co-receptors and ligands and initiates intracellular signaling. Inside the intracellular signal cascade, TLR4 is the only member of the TLRs family conveying the activating signal through two distinct intracellular signaling pathways, namely the myeloid differentiation protein 88 (MyD88) dependent and the TRIF (TIR-domain containing adaptor-inducing interferon-β)-dependent (13). In the MyD88-dependent pathway, MyD88 recruits a series of adaptor proteins that initiate signal transduction and activates the nuclear factor-κB (NF-κB) and mitogen-activated protein kinase (MAPK) pathways. These pathways induce transcription factor NF-κB and activator protein-1 (AP-1), thereby promoting production of several different inflammatory cytokines and chemokines, such as tumor necrosis factor-α (TNF-α), interleukin-1 (IL-1β), and IL-6, and monocyte chemoattractant protein-1 (MCP-1), among others (5, 13). Meanwhile, MyD88 downstream of other pathways, such as Ca^2+^/calmodulin-dependent protein kinase (CaMK II) (14, 15) and phosphatidylinositol 3-kinase/protein kinase B (PI3K/Akt) (16), can also be activated and promote inflammatory cytokine secretion dependent on the NF-κB transcription factor. Apart from the NF-κB and MAPK pathways, MyD88 has been shown to regulate other pathways, namely Ca^2+^/CaMK II and PI3K/Akt which regulate transcription of pro-inflammatory cytokines. In the TRIF-dependent pathway, the TLR4 extracellular complex is endocytosed into the cell, thereby allowing TRIF to migrate to receptors in the cytoplasm. TRIF promotes the activation of the transcription factor, in a similar fashion to the MyD88 dependent pathway, thereby inducing the expression of type I interferons (IFNs) and IFN-inducible genes (13, 17).

## TLR4 Initiates Inflammatory Response in Cardiac Hypertrophy

**Figure 1:**
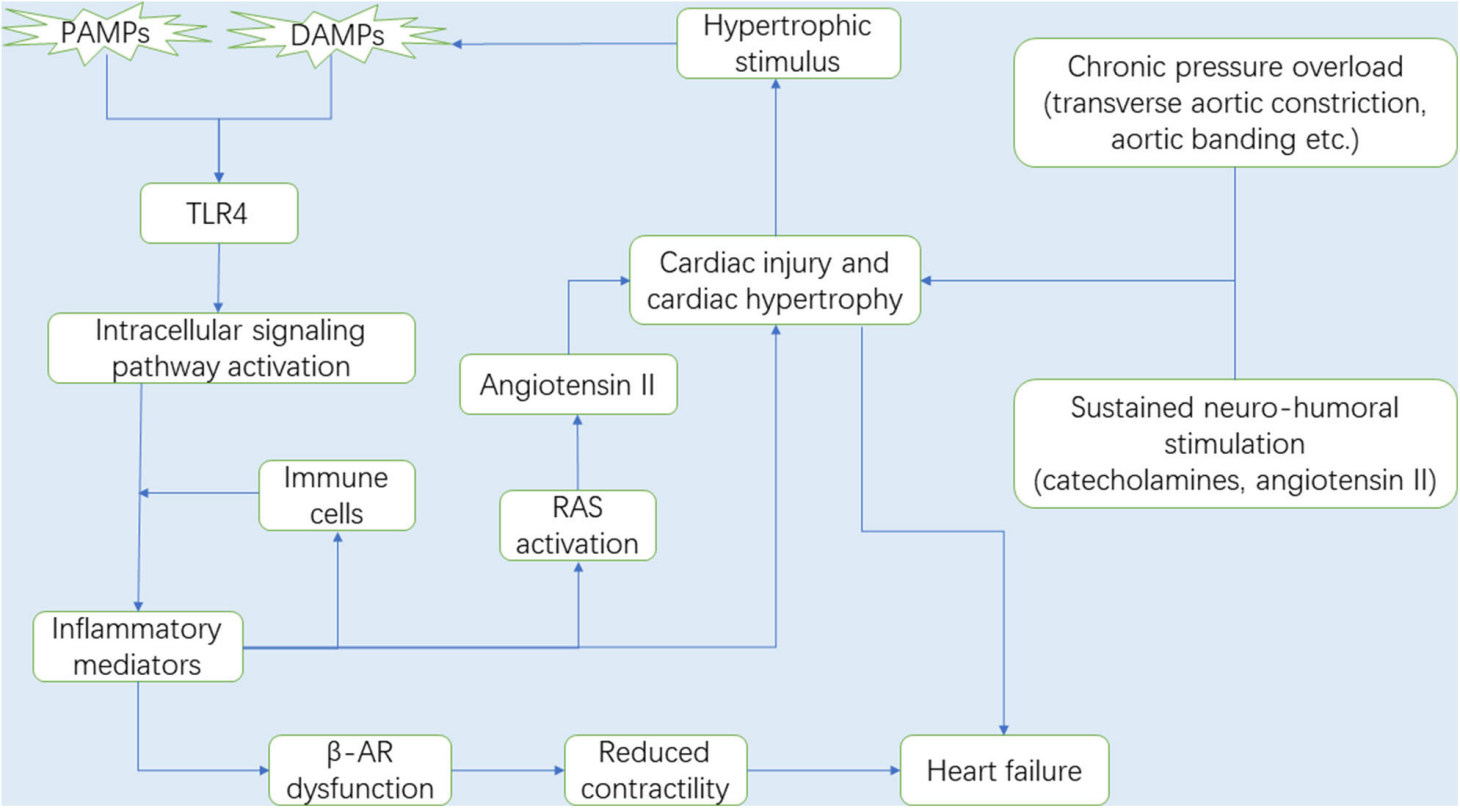
It is now well-known that the myocardium is gradually damaged and develops into cardiac hypertrophy under chronic pressure overload. During the process, RAS and the sympathetic-adrenomedullary system are activated. With the progression of cardiac hypertrophy, the necrotic myocardium releases DAMPs, in which DAMPs and some PAMPs activate TLR4, and TLR4 subsequently activates the intracellular signaling pathways that promote the secretion of inflammatory mediators (e.g., inflammatory cytokines and chemokines). Immune cells migrate from the circulation to the heart in response to chemokines and then secrete pro-hypertrophic cytokines (e.g., TNF-α, IL-1β, and IL-6). In this context, upregulated cytokines expression can not only lead to cardiac hypertrophy, but also promote Ang II production by activating RAS, which also accelerates the development of cardiac hypertrophy and gives rise to cardiac dysfunction. Interestingly, the injured myocardium in turn produces some hypertrophic stimulus that can be recognized by TLR4. Therefore, there is a circulatory inflammatory loop that accelerates cardiac hypertrophy. In addition, prolonged exposure cytokines, especially TNF-α, impair the sensitivity of the β-ARs to respond to β-agonist, suggesting a loss in β-AR responsiveness due to inflammation, and leading to cardiac contractile dysfunction and ultimately heart failure. Abbreviations: RAS, Renin-angiotensin system; DAMPs, Damaged associated molecular patterns; PAMPs, Pathogen associated molecular patterns; TLR4, Toll-like receptor 4; TNF-α, Tumor necrosis factor-α; IL-1β, Interleukin-1β; IL-6, Interleukin-6; β-AR, β-adrenergic receptor.

Mechanical overload and neuro-humoral stimulation, characteristic hallmarks of pathogenesis of cardiac hypertrophy, have been extensively studied (1, 2). Mechanical overload can be classified into pressure overload and volume overload. In fact, numerous studies have focused on pressure overload-induced cardiac hypertrophy (18), and the common surgical way includes transverse aortic constriction (TAC), aortic banding (AB) and abdominal aortic constriction (AAC), owing to a lack of an adequate volume overload mouse model that satisfactorily mimic the chronic course of valvular regurgitation in humans. Furthermore, the sustained neuro-hormonal activation leads to the generation of catecholamines and angiotensin II (Ang II) by the sympathetic-adrenomedullary system and the renin-angiotensin system (RAS), respectively, which contribute to ominous progression to hypertension and subsequent cardiac hypertrophy (19, 20). Hypertension is one of the most common pressure overload stimuli that induce cardiac hypertrophy and remodeling (21). Due to paucity of direct evidence, we speculate that long-term pressure overload exerts a mechanical force on the heart, thereby inducing release of hypertrophic stimuli, some of which act as DAMPs and activate TLR4 to mediate innate immune system and trigger inflammation (Figure 1).

Meanwhile, accumulating evidence suggests that TLR4 is an essential player in the progression of cardiac hypertrophy. It has been elucidated that TLR4 is associated with inflammatory response, and downregulation of TLR4 expression attenuates cardiac hypertrophy and prevents inflammation (22). This section discusses TLR4's function in cardiac hypertrophy.

Particularly, Ha et al. (23) initially described TLR4's role in cardiac hypertrophy using *in vivo* mouse model. Specifically, they applied AB in TLR4 deficient and wild type mice, and found that knocking out TLR4 reduces cell size and improves cardiac hypertrophy. On the other hand, Ehrentraut et al. (24) examined the role of TLR4 in cardiac hypertrophy but through pharmacological rather than knockout studies. Eritoran, a TLR4 antagonist that targets lipid A, is administered to C57BL/6 mice after TAC. Compared to the untreated groups, eritoran treated mice have a smaller left ventricular/body weight ratio. Quantitative real-time polymerase chain reaction and Enzyme-linked immunosorbent assay further revealed downregulation of hypertrophic markers and pro-inflammatory cytokines in drug-treated groups. Similarly, continuous subcutaneous infusion of Ang II increased the level of brain TLR4 in the Ang II-induced hypertensive rat model, activated myocardial inflammation and increased sympathetic activity, both of which are responsible for hypertension and cardiac hypertrophy. Conversely, central blockade of TLR4 reportedly reduced mean arterial blood pressure, suppressed production of pro-inflammatory mediators, and eventually attenuated cardiac hypertrophy (25, 26). Recently, blockade of TLR4 was found to display less hypertrophy in isoproterenol (ISO)-induced cardiac hypertrophy in rats (27).

As previously mentioned, TLR4 blockage exerts cardioprotective effects usually associated with inhibition of TLR4-mediated inflammation. On the contrary, a low dose of TLR4 agonist also produces the cardioprotective effects, and improves cardiac pressure overload-induced hypertrophy, possibly through stimulation of non-specific protective immune response by TLR4 agonist that protects against detrimental effects of pressure overload on the heart (28). Regardless of its blockage or activation, these studies strongly suggest that TLR4 is critical in the regulation of cardiac hypertrophy.

## The Role of TLR4 Co-receptors in Cardiac Hypertrophy

To explore TLR4 function intensively, researchers recently have shifted their attention to its co-receptors. In LPS/TLR4 signaling, TLR4 activation requires formation of a complex with its co-receptor called MD2, which is subsequently induced to dimerize to activate the TLR4 inflammatory cascade. TLR4's other co-receptors, such as LBP and CD14, are also involved in the dynamic process of LPS transferring to the TLR4/MD2 complex, prior to LPS interaction with TLR4 (11). Lipopolysaccharide (LPS) is a classical ligand that binds to LPS binding protein (LBP), the LBP/LPS complex attaches to another protein known as cluster of differentiation 14 (CD14), which catalyzes LPS transfer to another complex.

It has been shown that CD14 expression is increased in cardiac hypertrophy caused by TAC and further elevated after LPS stimulation (29). On the contrary, Shahini et al. (30) found that CD14 deficiency does not attenuate systolic blood pressure nor structure, function, or fibrosis within the myocardium, suggesting that its inhibition does not affect the maladaptive cardiac hypertrophy induced by Ang II. These contradictory results were clarified in the study by Han and colleagues who found that Ang II directly interacts with MD2 to facilitate the MD2/TLR4 complex formation, a process that is independent of LPS (31), it seems to explain why CD14 does not work in Ang II-induced cardiac hypertrophy. Thus, other molecules may also activate the TLR4/MD2 complex and cause inflammatory response via a mechanism similar to LPS. In Ang II-induced cardiac hypertrophy mouse model, MD2 deficiency was found to reduce cardiac inflammation as well as subsequent fibrosis, hypertrophy, and dysfunction by disrupting the combination of MD2 and TLR4 (31), supporting a mechanism by which Ang II activates TLR4 in an MD2-dependent manner. These findings were supported in a similar study where the obesity-induced cardiac hypertrophy model was investigated, in which a high-fat diet (HFD), such as palmitic acid, oxidized low-density lipoprotein, or total cholesterol, corresponding to lipids found at high circulating levels in hyperlipidemia, was used to induce cardiac hypertrophy model. Using a specific small molecule MD2 blocker L6H21 and MD2 knockout mice, researchers found that cardiac inflammation, fibrosis, and hypertrophy are mitigated in the HFD-induced obese mouse model by significantly reducing production of inflammatory cytokines (32, 33). So, the biological function and importance of MD2 are not limited to activating TLR4 signaling pathways by interacting with LPS, now with PA, cholesterol, and Ang II, having been demonstrated to bind to MD2. Furthermore, several studies have also demonstrated upregulation of TLR4 and MD2 in cardiac hypertrophy mice subjected to pressure overload (34, 35), with similar results also observed in myocardial ischemia/reperfusion injury (36). Therefore, MD2 may also be involved in pressure overload-induced cardiac hypertrophy, in a similar fashion to obesity- or Ang II-induced cardiac hypertrophy, although the underlying mechanism remains unclear.

Recently, these studies have revealed a novel molecule, Myeloid differentiation 1 (MD1). It is a highly homologous protein with MD2 and forms a complex with radioprotective 105 kDa protein (RP105) to antagonize TLR4-MD2 heterodimer, thereby inhibiting a downstream signaling cascade. On this basis, recent research demonstrated that MD1 expression is significantly reduced in HFD-induced obesity, which in turn leads to cardiac injury such as structural and electrical remodeling of the atrium (37), left ventricular (LV) hypertrophy, and fibrosis (38, 39). At the same time, cardiac injury was reportedly exacerbated under the obesity condition in mice that lacked MD1. Overall, these findings indicate that regulating expression of TLR4 co-receptor can be used as a therapeutic approach for treatment or prevention of TLR4-mediated inflammatory diseases.

## The Role of TLR4 Ligands in Cardiac Hypertrophy

TLR4 is a well-known PRR that recognizes its ligands from both PAMPs and DAMPs. It is now well-established that LPS as classical PAMP recognized by TLR4 is dependent on MD2. However, previous studies have shown that only some endogenous molecules from DAMPs such as high mobility group box 1 (HMGB1), heat-shock protein (HSP) 70, S100A8/S100A9 and fibronectin, among others, combine with TLR4 and trigger activation of inflammatory response through a specific interaction with TLR4/MD2 complex (40–43), while other ligands have not validated whether MD2 is essential for activation of TLR4 signaling pathways (44). During progression of cardiac hypertrophy, TLR4 ligands play crucial roles in modulating inflammatory response by binding to TLR4 (Table 1). An increase in TLR4 ligands pool accelerates progression of diseases such as hypertension or cardiac hypertrophy (27, 50), as well as common ligands such as HSPs, HMGB1, LPS, heparan sulfate (HS), fibrinogen, among others.

**Table 1:** Summary of the TLR4 signaling ligands in cardiac hypertrophy and cardiac remodeling.

**Ligands**	**Inducement**	**Downstream signaling**	**Effect**	***In vivo* or *in vitro***	**References**
HSP60	HSP60	TLR4/CaMK II	Promotes cardiomyocyte hypertrophy and initiated an inflammatory response	*In vitro*	(45)
HSP70	Abdominal aortic constriction	TLR4/MAPK	Promotes cardiac hypertrophy and fibrosis	*In vivo*	(46)
HMGB1	Chronic hypoxia	HMGB1/TLR4	Promotes experimental pulmonary hypertension and right ventricular hypertrophy	*In vivo*	(47)
HMGB1	Spontaneously hypertension	HMGB1/TLR4	Produces inflammatory alterations, sympathoexcitation, and cardiac hypertrophy	*In vivo*	(48)
Fibrinogen	Aortic banding or fibrinogen	TLR4/MyD88/NF-κB	Induces hypertrophic response of cardiac myocytes	*In vivo* and *in vitro*	(49)
HS	Spontaneously hypertension	HS/TLR4	Entails an aggravated cardiac inflammation and maintain hypertension	*In vivo*	(50)
Tenascin-C	A permanent ligation of the left anterior descending coronary artery	Tenascin-C/TLR4	Promotes shifting of the macrophage phenotype to a M1 phenotype and accelerates adverse ventricular remodeling after myocardial infarction	*In vivo*	(51)
FFA	High fat diet	TLR4/MD2/MAPK	Induces weight gain and cardiac hypertrophy, with increased cardiac fibrosis and inflammation	*In vivo*	(32)
Ang II	Ang II	TLR4/MD2/MyD88	Promotes cardiac inflammation and remodeling	*In vivo* and *in vitro*	(31)
LPS	Isoproterenol	TLR4/NF-κB	Induces oxidative stress and exaggerates the cardiac hypertrophy progression	*In vivo*	(27)

*TLR4, Toll-like receptor 4; HSP, Heat shock protein; HMGB1, High mobility group box-1; HS, Heparan sulfate; FFA, Free fatty acids; Ang II, Angiotensin II; LPS, Lipopolysaccharide; CaMK II, Ca^2+^/calmodulin-dependent protein kinase; MAPK, Mitogen-activated protein kinase; MyD88, Myeloid differentiation protein 88; NF-κB, Nuclear factor-κB; MD2, Myeloid differentiation protein 2*.

### Heat Shock Proteins

Heat shock proteins (HSPs), the most phylogenetically conserved proteins, constitute part of the molecular chaperone system of the cell. Heat shock protein family includes several members such as HSP60 and HSP70. However, their expression is upregulated and leaked into the extracellular compartment in response to exposure to stressful and/or damaged conditions, eventually as endogenous ligands inducing inflammatory response (52). Among all HSP members, HSP60 and HSP70 are the most important endogenous ligands to TLR4. The binding of HSPs (e.g., HSP60 and HSP70) with TLR4 leads to the recruitment of MyD88, activation of NF-κB, and increased expression of inflammatory cytokines such as tumor necrosis factor (TNF)-α, interleukin (IL)-1, and IL-6 and the release of chemokines by immune cells (53). Recently, Cai et al. (46) observed that the expression level of HSP70 is elevated in the myocardium and serum after pressure overload. However, the application of extracellular HSP70 antagonism in mice revealed that blockade of combining HSP70 with TLR4 decreases macrophage infiltration and inflammatory cytokine expression, thereby attenuating pressure overload-induced cardiac hypertrophy and fibrosis. Similarly, HSP60 was shown to be an agonist of TLR4, where it stimulates the innate immune system and induces cardiomyocyte hypertrophy and upregulates hypertrophic markers such as brain natriuretic peptide (BNP) and α-actin (45). In addition, it is interesting to note that the complement system is also activated by HSP60, as evidenced by its ability to induce inflammation at the adaptive immunity level that causes cardiac hypertrophy (45). Similarly, HSP60 was shown to exert cardioprotective and anti-apoptotic effects on animal models of myocardial ischemia/reperfusion injury (54), but HSP60 and TLR4 overexpression promote their interaction in hypertrophic myocardium, which may be a crucial event to permit harmful effects of HSP60 (55). Therefore, as discussed earlier, HSPs are important ligands that bind TLR4 and regulate production of inflammatory cytokines and the development of cardiac hypertrophy.

### High Mobility Group Box 1

High mobility group box 1 (HMGB1) is a nuclear protein widely found in eukaryotic cells. Its functions include regulating transcription, promoting DNA damage repair, stabilizing chromatin structure, and so on. HMGB1 can also be released from the nucleus or cytoplasm to the extracellular space through broken cell membranes when apoptosis or necrosis occurs. Extracellular HMGB1 is a typical DAMP that binds to TLR4 and regulates inflammatory responses (56). In a cardiac hypertrophy mouse model, myocardial HMGB1 protein expression and translocation from cytoplasm to nucleus were elevated, and overexpression of exogenous HMGB1 produced detrimental effects on myocardium, exacerbating cardiac hypertrophy and LV systolic dysfunction (57). Interestingly, cardiac hypertrophy and heart failure can be prevented when stable nuclear HMGB1 levels are maintained, it is due to HMGB1 in the nucleus attenuating DNA damage (58, 59). These studies indicate that HMGB1 may have dual functions in cardiac hypertrophy, depending on its subcellular localization. Previous studies have demonstrated upregulation of TLR4 mRNA and HMGB1 protein in cardiac hypertrophy mice following TAC (60). Additionally, Bauer and colleagues (47) elucidated the role for HMGB1/TLR4 interaction in driving right ventricular hypertrophy, suggesting that TLR4/HMGB1 axis is involved in the development of cardiac hypertrophy. These findings were corroborated by a subsequent study that examined HMGB1 levels. Particularly, administration of a specific TLR4 blocker, viral inhibitory peptide within the paraventricular nucleus, downregulated HMGB1 in both circulation and brain, and this was accompanied by downregulation of pro-inflammatory cytokines as well as improvement of cardiac hypertrophy (48). Another study investigated HMGB1 in H9c2 cells, and found that HMGB1 alone had no influence in cardiac hypertrophy but aggravated myocardial hypertrophy in the context of pressure/mechanical stress stimulation, which also caused a prompt upregulation of the expression of TLR4 in cardiomyocytes (61). Consequently, the TLR4/HMGB1 axis may be a new therapeutic target for cardiac hypertrophy.

### Fibrinogen

Fibrinogen, a coagulation factor produced by hepatic cells, is a plasma protein that plays a vital role in platelet aggregation and blood viscosity. However, under inflammatory stimulation, fibrinogen content increases and deposits in and around the blood vessels, exacerbating the inflammatory response. It was reported that fibrinogen interacts with TLR4, triggering the TLR4 signaling cascade, in a similar fashion to that of LPS and activating the downstream MAPK and NF-κB, and stimulating the expression of inflammatory cytokines as well as chemokines (62). On this basis, Li et al. (49) used *in vitro* experiments to demonstrate that fibrinogen stimulates hypertrophic response of cardiomyocytes by activating inflammation by binding with TLR4, which enlarges the size of cardiomyocytes and promotes protein synthesis. By using immunohistochemistry, they also showed that fibrinogen deposition in the myocardial extracellular matrix increased significantly following pressure overload, leading to homeostatic disruptions and cardiac dysfunction during the development of cardiac hypertrophy (49).

### Other Ligands

Heparan sulfate (HS), one of the major components of the extracellular matrix and plasma membranes, is also an endogenous ligand for TLR4. A combination between HS and TLR4 was found to enhance myocardial inflammatory reaction, thereby accelerating development of hypertension (50). Meanwhile, other TLR4 ligands, such as tenascin-C (63), hyaluronan (64), and S100 (42), are highly expressed in the hypertrophic myocardium, although it is not clear whether they regulate cardiac hypertrophy by binding to TLR4. Interestingly, TLR2 ligand lipoteichoic acid and TLR9 ligand CpG-oligodesoxynucleotide 1,668 thioate was found to induce hyperinflammatory cardiac response by binding to TLR4, and subsequently accelerating progression of cardiac hypertrophy, suggesting a selective or cross-regulation between TLRs and their endogenous ligands (22).

## TLR4 Signaling Pathways Associated With Cardiac Hypertrophy

**Figure 2:**
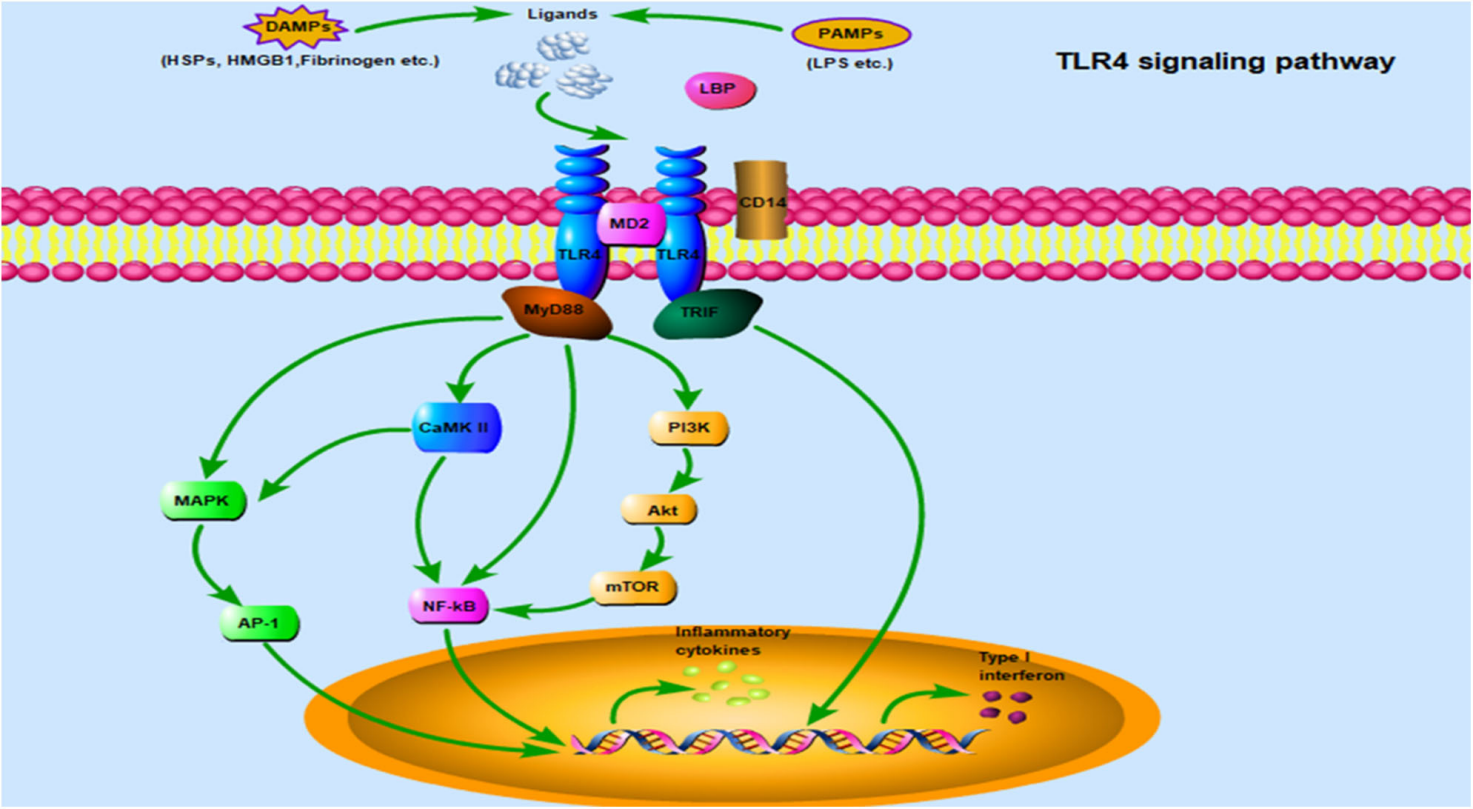
TLR4 signaling pathway: TLR4 is localized on the cell surface for ligand recognition, some of which are recognized after TLR4 interacting with MD2, even requiring CD14 and/or LBP involvement. Once activated by ligands on the cell surface, the TLR4/MD2 complex engage two separate downstream signaling pathways: the MyD88-dependent pathway and the TRIF-dependent pathway. The MyD88-dependent pathway mediates inflammatory response via some important transduction signaling such as NF-κB, PI3K/Akt, CaMK II, and MAPK, which promote NF-κB nuclear translocation and accelerate AP-1 transcriptional activation, and eventually leading to the secretion of inflammatory cytokines. In addition, there is crosstalk between MAPK and CaMK II signaling. TRIF plays an essential role in the MyD88-independent pathway through TLR4, type I IFNs are a crucial downstream molecule mediating hypertension and cardiac hypertrophy. Abbreviations: TLR4, Toll-like receptor 4; MD2, Myeloid differentiation protein 2; LBP, LPS binding protein; MyD88, Myeloid differentiation protein 88; TRIF, TIR-domain containing adaptor-inducing interferon-β; NF-κB, Nuclear factor-κB; PI3K/Akt, Phosphatidylinositol 3-kinase/Protein kinase B; CaMK II, Ca^2+^/calmodulin-dependent protein kinase; MAPK, Mitogen-activated protein kinase; AP-1, Activator protein-1; IFNs, Interferons.

Once TLR4 ligands interact with TLR4 on the cell surface, the signal can subsequently follow one of two distinct directions, namely the TLR4/MyD88 and TLR4/TRIF pathways. In fact, the NF-κB and MAPK pathways are common downstream pathways inMyD88-dependent signaling (65). Notably, the PI3K/Akt and Ca^2+^/CaMK II pathways have also been implicated in TLR4/MyD88 downstream signaling, where they play an important role in cardiac tissue damage (15, 65). Additionally, TRIF-dependent signaling is required for TLR4-mediated production of type I IFN. A comprehensive description of its mediated inflammatory response and host defense have been clarified in detail (17). This section mainly describes the elaborate network of TLR4-related signaling pathways during cardiac hypertrophy (Figure 2).

### TLR4/MyD88/NF-κB Pathway

Cardiac hypertrophy is closely associated with the TLR4/MyD88/NF-κB signaling pathway. Particularly, NF-κB is a downstream transcription factor in the TLR4-mediated signaling pathway that can be activated to enter the cell nucleus and promote expression of a series of pro-inflammatory cytokine genes as well as mediate the associated inflammatory response. Consequently, it acts as an important intracellular regulatory factor in the development of cardiac hypertrophy (66, 67). It was reported that inhibiting nuclear translocation of NF-κB can improve or block progression of cardiac hypertrophy (68, 69), and similar results were also observed in the hypertrophic model by the use of small interfering RNA mediated NF-κB silencing (67). MyD88 is the canonical adaptor for TLR4 downstream inflammatory pathways. Blockade of MyD88 reportedly exhibited beneficial effects in reducing hypertrophic response, whereas its overexpression activated the NF-κB pathway and subsequently contributed to cardiomyocyte apoptosis in pressure overload-induced cardiac hypertrophy *in vivo* (70, 71).

Apart from the classic pressure overload model, studies have reported other ways for inducing cardiac hypertrophy models. Importantly, activation of the TLR4/MyD88/NF-κB pathway was found in hypertrophic myocardium. For example, Trentin et al. (72) demonstrated that unilateral renal ischemia/reperfusion injury causing increased systemic inflammatory cytokines and TLR4 activity induces cardiac hypertrophy, and TLR4 knockout attenuates cardiac hypertrophy and electrical dysfunction. Moreover, chronic intermittent hypoxia, the most characteristic pathophysiological change of obstructive sleep apnea syndrome, has been reported to induce cardiac hypertrophy in animal models, and the beneficial cardiac effects are produced by inhibition of the TLR4/MyD88/NF-κB pathway (73). Up to date, a growing body of evidence has associated the TLR4/MyD88/NF-κB pathway within the brain with pathological hypertension, whereas RAS and sympathetic nervous system (SNS) are involved in regulating blood pressure. In fact, Ang II is a principal effector hormone of RAS, and plays an essential role in elevating levels of circulating plasma norepinephrine (NE, an indirect indicator of sympathetic activity) in hypertension and cardiac hypertrophy models. However, inhibition of TLR4 within the brain was reported to reduce NF-κB activity and downregulate inflammatory cytokines, and this was accompanied by attenuation of Ang II-induced hypertensive response and circulating NE levels (25). Moreover, inhibition of Ang II type 1 receptor or TLR4 blockade in the brain produced similar results in spontaneously hypertensive rats (48, 74). Overall, these results suggest that the TLR4/MyD88/NF-κB pathway is a bridge between RAS, SNS and hypertension, and provides a novel treatment therapeutic for hypertension and cardiac hypertrophy caused by hypertension. Furthermore, other mediators associated with the TLR4/MyD88/NF-κB signaling pathway have been identified. For example, insulin resistance is a well-known reciprocal cause of cardiac hypertrophy/heart failure and diabetes. A recent study showed that retinol-binding protein 4 (RBP4) might be an important node that mediates the vicious cycle of insulin resistance and cardiac hypertrophy/heart failure (75). In fact, its deficiency normalized the glucose transporter-4 (GLUT4) expression in cardiomyocytes and attenuated hypertrophic response to pressure overload, whereas TLR4 or MyD88 knockdown attenuated RBP4-induced insulin resistance and cardiac hypertrophy (75). Overall, these findings suggest that RBP4, through the TLR4-mediated signaling pathway, not only directly impair GLUT4 expression in cardiomyocytes but also promote expression of pro-inflammatory cytokines and cardiac hypertrophy (75). These studies suggest a strong association between TLR4/MyD88/NF-κB and cardiac hypertrophy.

### TLR4/MyD88/MAPK Pathway

The highly conserved MAPKs are serine-threonine kinases protein that play cardinal roles in the pathogenesis of various human diseases by coordinating diverse cellular activities such as cell differentiation, proliferation, survival, and inflammation (76). It consists of four well-characterized cascades, namely the extracellular signal-related kinases (ERK1/2), p38 MAPK, c-Jun N-terminal kinase (JNK), and ERK5. Functionally, TLR4 recruits adaptor proteins MyD88 activating MAPKs, which in turn modulate activation of several transcription factors, including AP-1, thereby contributing to expression of pro-inflammatory cytokines (77). Importantly, increased activity of almost all MAPKs components in myocardial tissues were found in the model of pressure overload-induced cardiac hypertrophy where they have diverse effects that jointly drive progression of cardiac hypertrophy (78). Meanwhile, MAPKs are well-established downstream signaling proteins in the TLR4 pathway and have been identified in cardiovascular disease. The TLR4/MyD88/ERK pathway's role in the heart has also been reported. For instance, LM8-mediated MyD88 inhibition reportedly exerts beneficial effects for cardiac hypertrophy and fibrosis by reducing inflammatory cytokine expression as well as immune cell infiltration in obese mouse models (79).

Numerous groups have studied the role of TLR4/MAPK signaling in cardiac hypertrophy using pharmacological inhibitors or genetic knockout mice, that directly or indirectly regulate ERK1/2, JNK, and p38 MAPK. In one study, interference with TLR4 expression was found to blunt ERK1/2 and p38 MAPK phosphorylation levels in spontaneously hypertensive rats, and subsequently improved vascular inflammatory response and cardiac hypertrophy (80). A recent study demonstrated that mice lacking MD2 (one of TLR4 co-receptor) were resistant to Ang II-induced cardiac hypertrophy in conjunction with reduced phosphorylation of ERK and subsequent cardiac inflammation, suggesting that TLR4/MD2/ERK signaling exerts pro-hypertrophic effects in the heart (31). In addition, inhibition of extracellular HSP70 (one of the TLR4 ligands) binding to TLR4 was found to attenuate pressure overload-induced cardiac hypertrophy and fibrosis by modulating ERK and p38 MAPK activity (46), further corroborating the previous studies. It is interesting to note that blocking TLR4 appeared to exert no effect on activation of ERK1/2 following stimulation of extracellular HMGB1 (one of the TLR4 ligand) in mechanical stress-induced cardiomyocyte hypertrophy *in vitro* (61), suggesting that various ligands may activate TLR4 downstream different pathways. Furthermore, a new regulatory mechanism for TLR4/MAPK signaling has been identified. Specifically, MD1 was downregulated in the hearts from patients with hypertrophic cardiomyopathy, whereas cardiac-specific overexpression of MD1 in mice exerted protective effects against obesity- or pressure overload-induced cardiac hypertrophy. In addition, recent research data showed that an interaction between MD1 and TLR4 prevents activation of MAPK signaling, thereby inhibiting hypertrophic response (35, 39). Thus, MD1 seems to be a novel pharmacological target for controlling development of cardiac hypertrophy. Additionally, a number of tyrosine kinases are activated when TLR4 is triggered. For example, RIP2 possesses tyrosine kinase activity that induces activation of MAPKs, with its deficiency has been found to control progression of cardiac hypertrophy following AB. The protective properties observed in RIP2 deletion mice subjected to pressure overload were attributed to inhibition of TLR4/MAPKs signaling, since RIP2 deletion mice lowered levels of ERK1/2, JNK, and p38 MAPK phosphorylation (81). Collectively, these data suggest that TLR4-mediated MAPKs activation plays an important role in cardiac hypertrophy.

### TLR4/MyD88/CaMK II Pathway

Another possible mechanism of cardiac hypertrophy is that TLR4 plays a role via the regulation of CaMK II, indicating that TLR4 downstream networks are more complicated than previously assumed and involve inflammasome activation or CaMK II participation. CaMK II belongs to a family of serine/threonine kinases that is abundantly expressed in cardiac tissues, and plays an important role in regulating cardiac structure and electrical activity. Upon activation, CaMK II acts as a downstream target that produces different effects such as inflammatory response and oxidative stress that promotes cardiac hypertrophy (82), and ion channels dysfunction that causes arrhythmias.

In a post-myocardial infarction hypertrophic model, CaMK II activation was found to promote expression of pro-inflammatory gene complement factor B (CFB) through the NF-κB pathway in both cardiomyocytes and mice (83), subsequently inducing cell membrane injury in cardiomyocytes. Furthermore, myocardial injury and inflammation after myocardial infarction are improved in mice with knockout CFB or CaMKII inhibition (83). Similarly, silencing of CaMK IIδB, one of the isoforms of CaMK II, protected against cardiomyocyte hypertrophy, and prevented CFB and NF-κB expression although it did not alter expression of inflammatory cytokines (45), indicating that CaMK II is indeed a key regulator of inflammatory response in TLR4 signaling. It has been reported that blocking MyD88, the downstream adapter protein for TLR4 signaling, significantly improves mortality and reduces oxidation-CaMK II expression and cardiac hypertrophy (15). Recently, several laboratory found that CaMK II as a downstream effector of TLR4 signaling (45, 83, 84) plays a key role in pathogenesis of cardiac hypertrophy under hyperlipidemia conditions. Additionally, mRNA levels of cell hypertrophic and fibrotic genes and endoplasmic reticulum stress markers were reversed by inhibition of TLR4 *in vitro* experimental models (84). In contrast, activation of the TLR4/MyD88 signaling pathway enhances CaMK II activity, thereby facilitating LV remodeling in the setting of chronic pressure overload or obesity to some extent (34, 38), illustrated that TLR4/MyD88/CaMK II pathway mediated inflammation involved hypertrophic remodeling. Moreover, the molecular mechanisms underlying CaMK II-mediated activation of inflammatory responses involved in obesity-induced cardiac hypertrophy indicates a crosstalk between CaMK II and cellular signaling cascades such as MAPKs and NF-κB signaling pathways (84).

On the other hand, CaMK II plays a critical role in regulating oxidative stress by disturbing the balance between oxidants and anti-oxidants in the heart, while its inhibition by either Myr-AIP or KN93 activates nuclear factor-like 2 signaling pathway promoting expression of anti-oxidant genes, such as heme oxygenase-1 and NADPH quinone acceptor oxidoreductase 1. This subsequently reduces ROS production in palmitate-treated H9c2 cells, thereby preventing cardiac remodeling (84).

Apart from its involvement in cardiac hypertrophy, CaMK II can also cause important changes in cellular electrical activity, leading to increase of vulnerability to arrhythmias. Under obesity/hyperlipidemia conditions, CaMK II activation causes downregulation of ion channels protein, such as Kv4.2, Kv4.3, Kv1.5, Kv2.1, and Cav1.2, and results in prolonged repolarization and action potential duration (APD), and eventually leads to detrimental remodeling of ion channels (38, 85). Furthermore, activation of CaMK II leads to impairment of intracellular Ca^2+^ homeostasis in HFD-induced mice (85). Overall, these observations support a related alteration of cardiac electrical activity mediated by TLR4/MyD88/CaMK II signaling. In summary, CaMK II as a node transducing upstream TLR4/MyD88 signaling plays a crucial regulatory role in cardiac structural and electrical remodeling.

### TLR4/MyD88/PI3K/Akt Pathway

The PI3K/Akt pathway has emerged as one of the most frequently activated drivers of cardiac hypertrophy. Activation of this signaling cascade was reported to be involved in physiological and pathological hypertrophy, leading to multiple changes, including alterations in cardiomyocyte morphology and survival, angiogenesis, and inflammatory cytokine secretion (86).

The regulation of the PI3K/Akt pathway by the growth hormone/insulin-like growth factor (IGF) axis has been associated with physiological hypertrophy and causes adaptive hypertrophy in the myocardium, which is manifested by increased cardiomyocyte size and angiogenesis, thus preserving cardiac function (87). Consistent with a protective role for PI3K/Akt pathway, it is evident that the activation of the PI3K/Akt pathway is considered as a negative feedback regulator of TLR4 signaling in cardiac inflammation to control and limit the pro-inflammatory process (65, 69, 77, 88). More recently, it was shown that erythropoietin exerts a protective role in myocardial fibrosis and cardiac hypertrophy, and the mechanism is associated with downregulating TLR4 expression by activating the PI3K/Akt signaling pathway (89). Notably, PI3K/Akt signaling also adversely affects the heart, possibly through the activation of PI3K different isoforms (p110α, p110γ). In other words, PI3K (p110α) stimulates adaptive cardiac hypertrophy, while PI3K (p110γ) regulates maladaptive cardiac hypertrophy (86). Besides, Oka et al. (90) illustrated in their review that the effect of Akt-mediated hypertrophic growth of cardiac myocytes (adaptive or maladaptive) depends on the duration of Akt activation. Some evidence has associated activation of PI3K/Akt signaling with pathological hypertrophy. For instance, Isorhamnetin, an anti-inflammation agent, was found to inhibit pressure overload-induced hypertrophic responses by the blockade of PI3K/Akt signaling (91). Furthermore, mammalian target of rapamycin (mTOR), as one of the direct downstream target proteins of the PI3K/Akt signaling pathway, has been found to enhance the pro-hypertrophic effects (92). Previously, it was reported that inhibition of mTOR by rapamycin attenuates pathological hypertrophic response by downregulating NF-κB signaling (93), whereas rapamycin has an additional improvement for cardiac hypertrophy in TLR4 deficient mice (23). On this basis, Li et al. (94) found that the upregulation of TLR4 reversed the protective effects generated by the suppression of the PI3K/Akt/mTOR pathway in Ang II-induced cardiomyocytes hypertrophy. Therefore, one of the mechanisms of cardiac hypertrophy is mediated by activation of the PI3K/Akt/mTOR signaling pathway under TLR4 stimulation, which brings a detrimental effect on the heart. Taken together, the role of PI3K/Akt signaling in cardiac diseases is not only associated with different PI3K isoforms and duration of Akt activation, but also with its upstream stimulator. However, whether the mechanism of TLR4/MyD88 in series of with PI3K/Akt signaling pathway regulates myocardial inflammation needs further exploration.

### TLR4/TRIF Pathway in Cardiac Hypertrophy

TLR4 transmits signals through the TRIF-dependent pathway, also known as the MyD88-independent pathway. It is considered as the primary signaling pathway responsible for mediating cardiovascular diseases as well. In fact, inhibition of TLR4/TRIF signaling cascade reportedly protected the heart in mice models of cardiac ischemia/reperfusion injury (95, 96).

Although comprehensive data is lacking, several studies have shown that the TRIF-dependent pathway exacerbates the hypertrophic response. It was reported that the TRIF-dependent pathway is shown to be a determinant in hypertension and cardiac hypertrophy, whereas the MyD88-dependent pathway is not responsible (97). Indeed, in settings of Ang II infusions at high rates (3,000 ng/kg/min), cardiac hypertrophy was found to have been reduced in TRIF deficient mice but aggravated in MyD88 knockout mice compared to wild type mice by measuring the ratio of heart weight to body weight, with a similar trend also observed in systolic blood pressure (97, 98). Furthermore, the inflammatory response not only did not decrease but increased in MyD88 deficient mice, accompanied by the increase in cardiac expression of TLR4 and TRIF, indicating that MyD88-dependent signaling functions as a negative regulator of pro-inflammatory pathological response mediated by TRIF in the context of high dose Ang II-induced cardiac hypertrophy (97), and infusion doses of Ang II may cause MyD88-dependent signaling functions in cardiac hypertrophy differing from the usual response. On this basis, further studies demonstrated that activation of TRIF signaling releases type I IFNs that are responsible for Ang II-induced hypertension and cardiac hypertrophy, whereas the TLR3-mediated TRIF signaling not only induces hypertension but also cardiac hypertrophy in response to Ang II infusion, but the TLR4/TRIF pathway is only required for cardiac hypertrophy (98). However, it is not clear whether there are similar phenomena in pressure overload- or obesity-induced cardiac hypertrophy model. As mentioned above, a recent study showed that LPS-RS, a TLR4 antagonist, reduces the expression of TRIF in the LV hypertrophy model caused by hyperoxia exposure, but not MyD88 (99). Collectively, these findings suggest that the TLR4/TRIF pathway is involved in cardiac hypertrophy.

## Effect of TLR4-Mediated Immune Cells in Cardiac Hypertrophy

**Figure 3:**
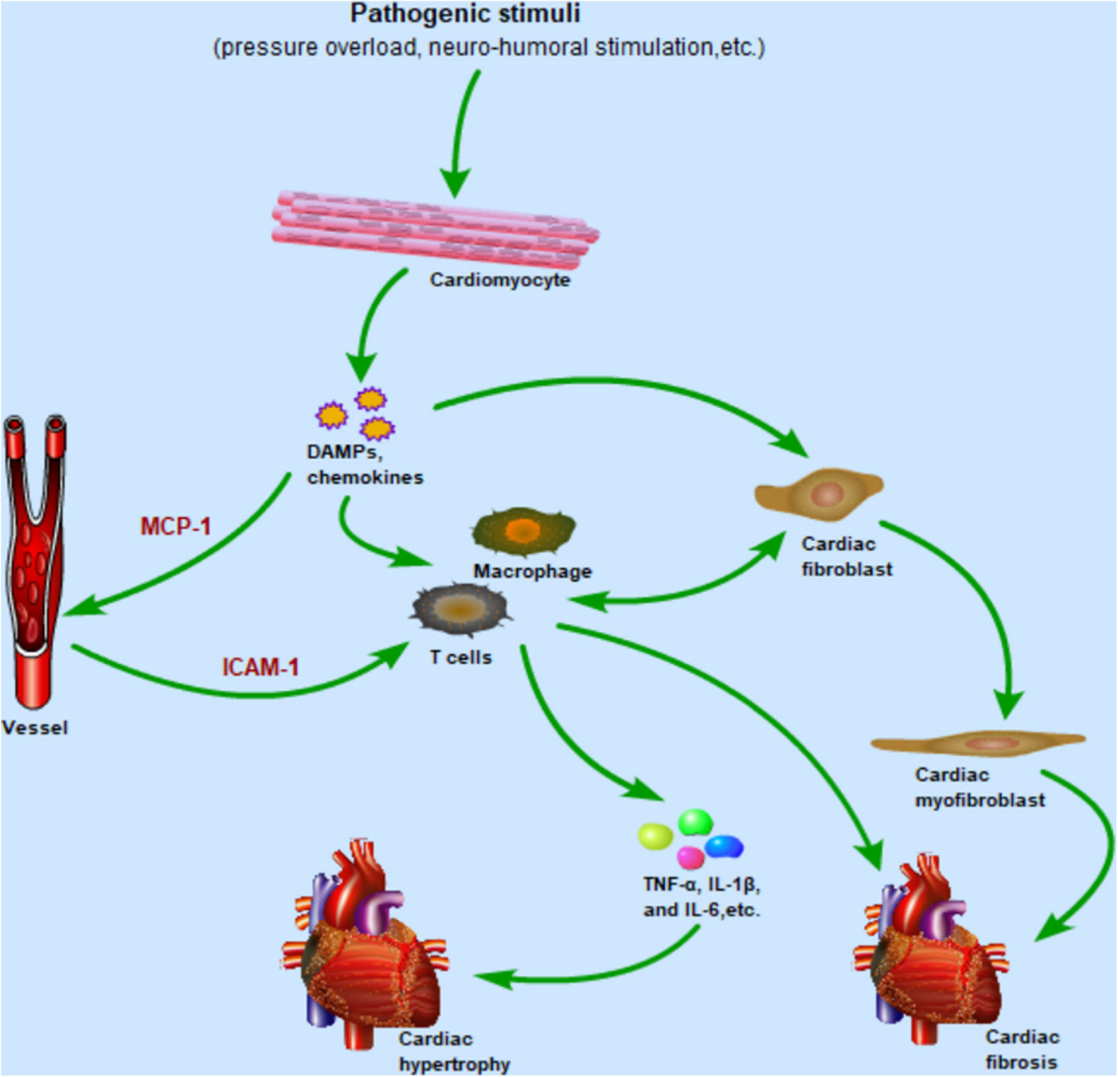
After cardiomyocytes are subjected to pathogenic stimulation, the TLR4 signaling pathway is activated to promote the secretion of chemokines and DAMPs, in which the upregulated expression of MCP-1 and ICAM-1 mediates the migration of macrophages and T cells from the circulation to the heart. Immune cells infiltrating the heart release inflammatory cytokines such as TNF-α, IL-1β, and IL-6, which promote the development of cardiac hypertrophy. Meanwhile, resident macrophages and fibroblasts are also activated by DAMPs and release inflammatory cytokines in the early stage of inflammation. As the inflammation enters the late stage, cardiac fibroblasts differentiate into cardiac myofibroblasts, mediating cardiac repair and fibrosis. Importantly, cardiac fibroblasts and immune cells communicate with each other to regulate pro-inflammatory and pro-fibrotic effects. Abbreviations: TLR4, Toll-like receptor 4; DAMPs, Damaged associated molecular patterns; MCP-1, Monocyte chemoattractant protein-1; ICAM-1, Intercellular adhesion molecule-1; TNF-α, Tumor necrosis factor-α; IL-1β, Interleukin-1β; IL-6, Interleukin-6.

TLR4 is widely expressed in many cells in the heart, such as cardiomyocytes, fibroblasts, endothelial cells, and various immune cells. Activation of TLR4-mediated inflammation pathways causes chemokine synthesis and secretion from host cells, which in turn promote immune cells migration to the heart and cardiac fibroblast differentiation. Subsequently, these immune cells generate inflammatory cytokines to orchestrate complex pathological response of cardiac hypertrophy. Meanwhile, cardiac fibroblasts are also involved in cardiac inflammatory responses by producing numerous cytokines and communicating with immune cells (Figure 3). The phenotypic changes of immune cells are associated with their function in the heart. In this part, more attention will be paid to the function of macrophages, T cells, and cardiac fibroblasts in cardiac hypertrophy and cardiac remodeling.

### Cardiomyocytes

TLR4 is the most abundant member of the TLRs family identified in human cardiomyocytes, and activated TLR4 signaling has been reported to promote secretion of cytokines and DAMPs in the myocardium in response to pathogenic stimuli (100, 101). These inflammatory mediators from cardiomyocytes induce activation and expansion of immune cells and cardiac fibroblasts, which then secrete both pro-inflammatory and pro-fibrotic cytokines to induce cardiomyocyte hypertrophy, cardiac fibroblast differentiation, and immune response (102).

### Macrophages

During cardiac hypertrophy, the cardiac tissue undergoes recruitment and infiltration of macrophages, a phenomenon that can be attributed to activation of TLR4 signaling, and the degree of infiltration of monocytes/macrophages and cytokine expression in macrophages are correlated with cardiac hypertrophy (28, 32, 46, 99, 103). However, cardiac macrophages function more extensively than previously thought, playing different roles in regulating cardiac hypertrophy and remodeling.

Macrophages can be divided into two phenotypes, namely the classical M1 and alternative M2, based on their functional, as well as activation by T helper cell (Th) 1 and Th2-mediated cytokines. This represents two extremes of macrophage activation state changes. Particularly, the M2 phenotype has been implicated in restricting these inflammatory response and mediating tissue repair, whereas the M1 phenotype secretes large amounts of pro-inflammatory mediators and contributes to inflammation. Consequently, manipulation of the macrophage phenotypes may provide a more advantageous way to prevent specific deleterious inflammatory effects than depletion or blockage macrophage responses, while still allowing for other critical phagocytosis and repair response. Recently, several studies have demonstrated occurrence of maladaptive cardiac remodeling that preserve the cardiac architecture following Doxorubicin treatments. In fact, M1 pro-inflammatory macrophages and cytokines were elevated whereas a concomitant decrease of M2 anti-inflammatory macrophages was reported in Doxorubicin-induced cardiomyopathy models (104, 105). Administration of embryonic stem cell-derived exosomes revealed curative effects on adverse cardiac remodeling in Doxorubicin-induced cardiomyopathy by regulating the balance between M1/M2 macrophages (104), whereas mesenchymal stromal cells-derived exosomes produce myocardium healing following myocardial ischemia/reperfusion injury by shifting polarization of macrophages favorable to M2 other than M1 phenotype (106). However, few studies have focused on the role of the phenotype of functional macrophages in progression of pressure overload and Ang II-induced hypertrophic remodeling.

It is interesting to note that TLR4 may be a potential target for regulating macrophage polarization. During cardiac remodeling, the predominance of different macrophage phenotypes is age dependent. Modulation of M1 macrophage polarization and the HMGB1/TLR4 cascade will eventually lead to reduction of the M2 phenotype as well as myocardial dysfunction during aging (107). Meanwhile, Tenascin-C is a TLR4 ligand that promotes polarization of M1, but inhibits M2 macrophage through TLR4, thereby accelerating adverse ventricular remodeling after myocardial infarction (51). In addition, it should take into account the fact that M1 macrophage dominates early stages of disease occurrence, whereas M2 macrophage predominate during the later stages (108). Consequently, earlier phenotype transformation of M1 to M2 macrophages has resulted in prominent improvements in myocardial infarction wound healing and cardiac function (109, 110). More importantly, macrophage polarization has been associated with the TLR4-mediated inflammatory pathway. In fact, inhibition of TLR4 signaling was found to accelerate the M1 to M2 phenotype polarization transition at Day 3 and subsequently improve cardiac function at Day 7, thereby reducing adverse LV remodeling (110). Hence, administration of therapies targeting macrophage polarization is also time-dependent.

### T Cells

Under pressure overload stimulation, increased cytokines in the cardiac microenvironment and upregulated cell adhesion molecules can facilitate T cell migration and infiltration into the heart. This in turn causes cardiac hypertrophy and heart failure as reviewed by Liu et al. (111). In addition, the TLRs signaling pathway may be responsible for secretion of inflammatory mediators, regulation of naïve T cells differentiation as well as bridging innate and adaptive immunity (111, 112). Numerous studies have also demonstrated the pivotal role played by T cells in stimulation of cardiac hypertrophy. Recombination activating gene 2 (RAG2) deletion mice were found to be completely deficient in mature B and T lymphocytes, as RAG2 is necessary for the generation of immunoglobulins and T cell receptors (TCR). Importantly, mice with genetic depletion of RAG2 manifested improved cardiac contractile function and prevented cardiac dilation but not ventricular hypertrophy induced by pressure overload (113). Similarly, TCR deficient mice lacking effective T cells or inhibited T cell co-stimulation exhibited beneficial effects for cardiac hypertrophy and heart failure following TAC, and their protective effects were closely related to inhibition of T cell activation (114, 115), emphasizing the dominant role of T cells in the progression of cardiac hypertrophy and heart failure. Moreover, T cells mineralocorticoid receptor knockout mice failed to develop cardiac hypertrophy and heart failure under pressure overload stimulation, and this was accompanied by repressed cardiac inflammatory response (116). T cells mainly comprise CD8+ T and CD4+ T cells, with CD4+ T cells playing a more critical role in cardiac hypertrophy (113, 116). Moreover, CD4+ T cells can be further classified into many subpopulations, such as regulatory T cells (Treg cells) and Th cells, among which Th cells include Th1, Th2, and Th17 cells. At present, numerous literatures have demonstrated that Treg cells play a protective role in cardiovascular diseases such as hypertension, cardiac hypertrophy, and myocardial infarction (109, 117). Moreover, Th1 and Th2 cells, as mentioned above, may be potential targets for regulating macrophage phenotypes. Particularly, Th1 exhibits pro-inflammatory effects in many inflammatory diseases, whereas Th2 plays an anti-inflammatory role, so the progression of inflammatory diseases can be regulated by manipulating the balance of Th1/Th2(or Th1/Th2 cytokine ratio) (118). Importantly, IFN-γ is upregulated but IL-4 is downregulated in heart mediastinal draining lymph nodes in mice following TAC. Since IFN-γ and IL-4 are critical hallmarks for Th1 and Th2, respectively, suggesting that T cells tend to Th1 polarization in pressure overload-induced cardiac hypertrophy (113). Collectively, in addition to maintaining the proportion of Treg cells among the CD4+ T cells or enhance their immunosuppressive functions, modulating Th1/Th2 (or Th1/Th2 cytokines ratio) balance may also be an effective strategy to regulate cardiac hypertrophy and suppress inflammatory response.

Emerging evidence has further demonstrated that other immune cells, such as neutrophils and dendritic cells, play a significant and direct role in promoting cardiac inflammation and progression of cardiac hypertrophy, but until recently, there has been limited data on the relationship between TLR4 signaling and neutrophils, dendritic cells during development of cardiac hypertrophy and remodeling.

### Cardiac Fibroblasts and Cardiac Myofibroblasts

Apart from immune cells, cardiac fibroblasts, one of the most common cell types in the heart, also play a significant role in cardiac inflammation. They act as sentinel cells expressed pattern recognition receptors TLR4 and are activated by PAMPs and DAMPs, secreting a number of chemokines and cytokines (119). Meanwhile, cardiac fibroblasts can differentiate into cardiac myofibroblasts, promoting cardiac tissue wound healing (120). A recent study found that TLR4 is involved in the differentiation process of cardiac fibroblasts. In the early stage of inflammation, LPS treatment was found to induce TLR4 activation, which prevented differentiation of cardiac fibroblasts and downregulated α-smooth muscle actin (α-SMA) expression, a marker of cardiac myofibroblasts (121). However, cardiac fibroblasts with pro-inflammatory effects are stimulated by LPS to differentiate into cardiac myofibroblasts with pro-fibrosis function through autophagy during tissue reconstruction after myocardial infarction (122).

Furthermore, TLR4 is not only expressed in cardiac fibroblasts, but also in cardiac myofibroblasts. A recent study showed that Tenascin C activates TLR4 in human cardiac myofibroblasts, inducing production of IL-6 and resulting in maladaptive cardiac remodeling (123). Similarly, LPS treatment was found to induce TLR4 activation, regulating synthesis and release of IL-1β in both cardiac fibroblasts and cardiac myofibroblasts (124). However, IL-1β secreted by cardiac fibroblasts maintained the early inflammatory response, while IL-1β released by cardiac myofibroblasts mediated the later inflammatory stages to promote wound healing (124).

It is worth noting that cardiac fibroblasts have been shown to communicate with immune cells in the heart (125). Under the stimulation of TLR4 ligands such as LPS and DAMPs, cardiac fibroblasts can promote the transformation of monocyte phenotype into pro-inflammatory M1 macrophages, while under the stimulation of transforming growth factor-β1 (TGF-β1), cardiac fibroblasts can direct monocytes toward anti-inflammatory/pro-fibrotic M2 macrophages (126). Interestingly, monocytes can adhere to cardiac fibroblasts and trigger secretion of TNF-α and TGF-β to transform fibroblasts to myofibroblasts, which also is relevant to the activation of TLR4 (127).

## Effect of TLR4-Mediated Inflammatory Mediators in Cardiac Hypertrophy

Cardiac hypertrophy is accompanied by activation of TLR4-mediated inflammatory pathways, which subsequently induce production of hypertrophic markers, chemokines, adhesion molecules, and a large number of pro-inflammatory cytokines (Figure 3). These have diverse effects on cardiac inflammation.

### Natriuretic Peptides

Natriuretic peptides (NPs) have been extensively studied as a diagnostic biomarker of heart failure, but also as a hypertrophic marker in cardiac hypertrophy. They include atrial natriuretic peptide (ANP) and B-type natriuretic peptide (BNP), which are induced by pathological stimulation and are synthesized mainly in cardiac tissues, causing a series of biological effects such as diuresis, natriuresis, vasorelaxation, thus serving as protective molecules of the heart (128). Meanwhile, these peptides have been shown to stimulate production of cyclic guanosine monophosphate and protein kinases G by interacting with the natriuretic peptide receptor, showing the anti-hypertrophic effects in the cardiac hypertrophy model (128, 129). Due to NPs' beneficial effects, Nesiritide, a synthetic BNP, has been applied to patients with acute decompensated heart failure and has achieved sound therapeutic efficacy (130). As a consequence, modulation of NPs may represent a useful strategy for developing strategies for prevention and treatment of cardiac hypertrophy.

### Monocyte Chemoattractant Protein-1 and Intercellular Adhesion Molecule-1

Monocyte chemoattractant protein-1 (MCP-1), also known as chemokine ligand 2, plays a crucial role in guiding migration of immune cells and promoting leukocytes infiltration. Functionally, MCP-1 binds to its receptor, CC chemokine receptor 2, to alleviate inflammatory disease (131). However, high levels of MCP-1 expression have been detected in the Ang II-induced cardiac hypertrophy model, exhibiting a deleterious role for MCP-1 in cardiac dysfunction and hypertrophy (103). In contrast, inhibition of MCP-1 was found to significantly prevent cardiac fibrosis and appearance of fibroblasts in response to Ang II infusion (132). Although no significant differences were observed between wild type and MCP-1 knockout mice in over a 6-week course of Ang II infusion in cardiac hypertrophy, MCP-1 deficiency suppressed inflammatory cytokine production and reduced fibrosis during early stages (132). In addition, clinical studies have revealed no difference in MCP-1 levels in patients with resistant vs. mild-to-moderate hypertension, but lower MCP-1 levels were recorded in hypertensive patients with LV hypertrophy (133). These findings suggest a possible downregulation of MCP-1 levels in hypertensive patients with advanced stage of cardiac damage. In addition, during MCP-1 guidance of leukocyte migration, another cytokine, intercellular adhesion molecule-1 (ICAM-1), seems to be of particular importance in mediating leukocyte adhesion to vascular endothelium followed by their migration into sites of inflammation. Specifically, ICAM-1 belongs to a member of an immunoglobulin-like superfamily of adhesion molecules capable of being expressed on the cell surface and plays a crucial role in the pathogenesis of cardiac hypertrophy (134). Recent studies found that the primary mechanism of ICAM-1 mediated cardiac remodeling induced by Ang II involves ICAM-1's combination with lymphocyte function-associated antigen-1 on the cell surface, which promotes monocyte/macrophage adhesion to vascular endothelial cells and their subsequent migration into the vessel and cardiac tissues (135). These changes induce production of pro-inflammatory cytokines such as TNF-α, IL-1β, and IL-6, leading to hypertension and cardiac remodeling (135, 136). However, treatment with an anti-ICAM-1 neutralizing antibody significantly prevents myocardial fibrosis without affecting arterial pressure and myocyte hypertrophy in pressure-overloaded hearts (134). Thus, further investigation is needed to clarify the role of ICAM-1 in hypertrophic remodeling.

### Inflammatory Cytokines

Cytokines have been reported to exert a marked influence on the cardiovascular system. For example, some inflammatory cytokines such as TNF-α, IL-1β, and IL-6 directly induce cardiac hypertrophy and correlated with the severity of hypertrophy. TNF-α plays a central role in initiating and sustaining the pro-inflammatory cytokine cascade that in turn stimulates production of other cytokines, such as IL-1β and IL-6. TNF-α, IL-1β, and IL-6 all have direct pro-hypertrophic effect on myocardial cells. Another mechanism by which these inflammatory cytokines are involved in cardiac hypertrophy and heart failure is associated with RAS and the adrenergic system (137–139) (Figure 1).

TNF-α, mainly generated by activated macrophages, is a ubiquitous inflammatory cytokine that regulates the pathological process of cardiac hypertrophy. Under either pressure or volume overload model, upregulated TNF-α expression has been associated with cardiac hypertrophy, especially in pressure overload-induced hypertrophy (18). A previous study found that global knockout of TNF-α ameliorates pressure overload-induced cardiac hypertrophy and cardiac dysfunction (140). In contrast, cardiomyocyte hypertrophy is accompanied by increases of ANP when neonatal rat ventricular myocytes are exposed to TNF-α (141). However, Ginsenoside Rg1, a traditional Chinese medicine, was found to downregulate TNF-α expression, both at mRNA and protein levels, in a concentration-dependent manner in rat hearts following pressure overload-induced cardiac hypertrophy (141). Moreover, Ang II-mediated ROS production and NADPH subunit activation were prevented by TNF-α deficiency, thereby improving hypertensive and hypertrophic response (142). Conversely, Ang II-induced hypertension and cardiac hypertrophy were gradually aggravated, due to TNF-α-mediated intracellular signaling (142).

IL-1β, the most studied member of the IL-1 family, has been associated with development of cardiac hypertrophy. In support of having a detrimental role during cardiac hypertrophy, IL-1β deficient mice undergoing pressure overload exhibited improved cardiac function and reduced cardiac hypertrophy, indicating that IL-1β promotes pathological hypertrophy (143). Another study further showed that activation of caspase-1-dependent pyroptosis plays a role in the pathogenesis of cardiac hypertrophy, especially its activation upregulated IL-1β expression and promoted IL-1β transformation to the active state (144). In contrast, phytopharmaceuticals, such as asiatic acid, not only repressed IL-1β-induced cardiomyocyte hypertrophic response, but also suppressed upregulation of IL-1β as well as TAC-induced cardiac hypertrophy (145). Consequently, downregulation of IL-1β and/or inhibition of its bioactivity in the heart are generally considered to be a certain cardioprotective intervention against cardiac hypertrophy.

As a cytokine with multiple physiological effects, IL-6 plays a significant role in diverse cells and organs, especially the myocardium. During myocyte hypertrophy, genetic deletion of IL-6 has been shown to ameliorate cardiac dysfunction and suppress Ang II-induced cardiac hypertrophy, suggesting that it has an exacerbating role in hypertrophic remodeling (146). On the contrary, cardiac hypertrophy was not improved in IL-6 knockout mice under Ang II and high salt stimulation (147). However, these two studies have in common that IL-6 absence alleviates inflammation by reducing myocardial macrophage infiltration during Ang II-induced cardiac injury (146, 147), indicating that IL-6 is related to Ang II-induced inflammation. However, IL-6's role in cardiac hypertrophy induced by pressure overload remains controversial (148, 149), possibly due to differences in the severity of TAC and in follow-up durations. These conclusions indicate that IL-6 plays a complex role in various stimulation-induced cardiac hypertrophy, and the function of IL-6 in cardiac hypertrophy remains to be investigated.

## Targeting TLR4 Signaling in Cardiac Inflammation: Potential Therapeutic Approaches

Currently, only a handful of treatment options have been developed for cardiac hypertrophy. These approaches, mainly involving lowering of blood pressure and improving cardiac function, have not effectively lowered the associated high mortality rate. Thus, it is necessary to identify new strategies that can effectively manage pathophysiology of cardiac hypertrophy. Since the TLR4 signaling pathway plays a significant role in many inflammatory diseases and is related to their pathogenesis, targeting it may be a potent strategy for controlling excessive inflammation. Particularly, targeting TLR4 signaling using molecules that directly bind to TLR4 or the TLR4/MD2 complex, and participate in ligands transferring or TLR4 intracellular signaling activation, modulating of immune cells polarization and inflammatory mediator production may be a feasible approach. However, the improvement or treatment of cardiac diseases with TLR4 inhibitors to date have not been launched in clinical trials, possibly because TLR4 inhibitors such as TAK-242 and eritoran have shown inadequate efficacy in other systems or organs.

Recently, Fujiwara and colleagues (150) used nanoparticle technology to deliver TAK-242 into monocytes/macrophages during myocardial reperfusion, and found a significant reduction in infarct size. Meanwhile, the concentrations of TAK-242 in the heart is found to be 90-fold higher in the injection of nanoparticles containing TAK-242 compared to the injection of the TAK-242 solution, suggesting that nanoparticles can effectively deliver the drug to the heart (150). Interestingly, nanoparticles are not only drug carrier systems, but also anti-cardiac hypertrophy agents that inhibit ISO-induced cardiac hypertrophy (151). Thus, nanoparticle technology may be a novel and clinically feasible strategy for managing heart diseases.

Moreover, studies have used some small molecule inhibitors to target TLR4 signaling and found promising outcomes in animal models. For example, Carvedilol, a classic anti-heart failure drug, reportedly exhibited potent TLR4 inhibition activity. The most efficient compound, 8a, was found to bind to LPS/TLR4/MD2 complex and generate strong anti-inflammation effects (152). Similarly, T5342126 was found to act as a small molecule TLR4 inhibitor, presumably functioning by selectively disrupting the mechanism of TLR4/MD2 interaction (153). Another novel TLR4 antagonist, Ibudilast, effectively suppressed production of malondialdehyde and TNF-α, and protected against cardiac dysfunction (154). It is worth noting that the degradation of activated TLR4 by ubiquitination was shown to attenuate pressure overload-induced cardiac hypertrophy and improve cardiac function, indicating that it may be an important approach for preventing progression of cardiac hypertrophy (155).

Another alternative therapeutic strategy is blocking TLR4 upstream DAMPs to diminish inflammation. For example, injection of box A, an inhibitory domain HMGB1, into the heart can reverse cardiac hypertrophy and delay heart failure induced by pressure overload (57). Apart from these, more attention should be paid to dynamic change and functional phenotypes of immune cells (102). Overall, further preclinical and clinical studies are needed to explore the therapeutic property of targeting of the TLR4 signaling.

## Conclusions and Future Perspectives

Inflammation has been widely accepted to play an important role in the pathological mechanisms of cardiac hypertrophy. Excessive chronic myocardial inflammation reportedly induces maladaptive myocardium growth and causes heart failure. In this review, we have described recent advances elaborating on the role of TLR4 signaling in cardiac hypertrophy. In summary, TLR4 interacts with its ligands and co-receptors to initiate expression of a number of inflammatory mediators, induce immune cell infiltration in the heart, and act as an important mechanism for activating various intracellular inflammatory signaling.

However, current knowledge on the role of TLR4 signaling remains insufficient. For instance, [1] the specific mechanism of TLR4 activation remains unanswered, the mechanism that more ligands bind to TLR4 should be further explored in the hypertrophic heart; [2] further studies are needed to elucidate the mechanism underlying the TLR4/TRIF pathway's action in cardiac hypertrophy; and [3] immune cell phenotypes and their corresponding functions during development of cardiac hypertrophy remains to be explored. Exploring these areas is expected to potentially reveal multiple targets for preventing or arresting development and progression of cardiac hypertrophy. Future studies are expected to focus on the following; [1] identifying more efficient TLR4 inhibitions capable of blocking TLR4 signaling activation; [2] unraveling the specific molecules regulating TLR4 downstream signaling pathways; [3] identifying novel TLR4 signaling upstream molecules; and [4] Using new technology to facilitate drug delivery specifically to heart. Therefore, TLR4 inhibitors completely transition toward clinical application that needs further study in animal models.

## Author Contributions

HH organized the article. ZX and BK wrote the draft. CD, JF, and TQ participated in the conception, discussion, and revision of the draft. HY edited the language and figure. All authors contributed to the article and approved the submitted version.

## Conflict of Interest

The authors declare that the research was conducted in the absence of any commercial or financial relationships that could be construed as a potential conflict of interest.
